# Putting patients first: Today's disparities research leading to health equity tomorrow

**DOI:** 10.1111/1475-6773.13107

**Published:** 2019-01-21

**Authors:** Cara V. James

**Affiliations:** ^1^ Office of Minority Health Centers for Medicare & Medicaid Services Baltimore Maryland

The Centers for Medicare & Medicaid Services (CMS) has collaborated with a wide variety of stakeholders to support work in all three areas of its path to equity: increasing the understanding and awareness of disparities and their causes, developing and disseminating solutions, and implementing sustainable actions. To increase understanding and awareness of disparities, CMS sponsored this issue, with a goal of contributing to the conversation on health disparities and emphasizing the value of continuing research in this area. The studies included in this issue underscore the importance of identifying groups of people who do not benefit equally from our health system and identifying root causes of these differences. We value the information and analysis they provide on this important topic and hope that they will create further discussion on how to reduce health disparities.

Sponsoring this issue is only the latest in a series of things CMS has done to improve health equity nationwide. To improve our understanding of disparities and their causes, we have fostered demographic data collection through the implementation of data standards; analyzed and reported on health disparities through annual reports on health care quality in Medicare Advantage and other analytic reports; and launched the Mapping Medicare Disparities Tool, which is an interactive web‐based tool that allows the user to quickly calculate a range of health outcome measures by population of interest at the county, state, and national levels.

The agency also launched the CMS Equity Plan for Improving Quality in Medicare, which focuses on six priority areas, such as increasing the ability of the health care workforce to meet the needs of vulnerable populations, improving physical accessibility of health care facilities, and improving communication and language access for individuals with limited English proficiency and persons with disabilities. We have also implemented *From Coverage to Care* (C2C)*,* an initiative to help individuals understand their coverage and how to use it to connect to the primary care and preventive services that are right for them; released the first ever *CMS Rural Health Strategy*; developed a number of resources to help stakeholders build an organizational response to health disparities; and provided Health Equity Technical Assistance to organizations seeking help to identify and address health disparities.

Finally, we worked across the agency to ensure that all of our programs are looking for ways to incorporate a focus on health equity, such as the Quality Improvement Organization Program, which seeks in part to improve health quality at the community level for all Medicare beneficiaries; the Partnership for Patients initiative, a public‐private partnership that aims to improve quality, safety, and affordability of health care; and models such as the Accountable Health Communities Model, a 5‐year model that tests whether systematically identifying and addressing the health‐related social needs of Medicare and Medicaid beneficiaries, such as food insecurity and inadequate or unstable housing, through screening, referral, and community navigation services will impact health care costs and reduce health care utilization. We also finalized a payment code for providers who spend additional time during a visit with patients who need it, including persons with a disability or a cognitive impairment.

Our efforts are a great start, but we know there are more to do and more to learn, which is why CMS’ Office of Minority Health (OMH) is pleased to support this Special Issue of Health Services Research. This issue further evaluates several areas of interest to CMS, such as chronic disease, quality of care, patient experience and satisfaction, and health coverage. The authors conducted innovative research to examine the multitude of influences that impact health disparities and promote innovation in quality improvement programs, and the targeted interventions to support the most vulnerable populations. Taken together, they move us a little closer toward our goals of achieving health equity.

This Special Issue begins with three manuscripts that examine chronic disease through a health equity lens. The first manuscript by Durfey et al (2019) uses Area Deprivation Index (ADI) measures to explore chronic disease management among Medicare Advantage enrollees. While the authors suggest that ADI has limitations as a measure of a social determinant of health, they also suggest that it may help Medicare assess individual risk and target interventions where MA enrollees live. They found that area deprivation is a predictor of chronic disease management and that the relationship did not differ by race or ethnicity. This association remained statistically significant after controlling for individual‐level risk factors. Only the top 10 percent most disadvantaged neighborhoods had a significant association with blood pressure control after adjustment. The authors suggest this may indicate the need for prioritization of resources to that segment of the population.

Another aspect of place, living in a “food swamp,” is the focus of the next manuscript. Phillips and Rodriguez (2019) note that while this term is relatively new to the public health literature, food swamps are places in which large numbers of unhealthy energy‐dense food offerings inundate, or “swamp out,” the relatively few existing healthy food offerings. They note the contrast with a “food desert,” which is defined more by a paucity of healthy options. The authors combine multiple data sources to complete a cross‐sectional analysis of 784 counties across 15 states. The study found a positive association between food swamp score and all‐cause hospitalizations with a stronger association in rural counties than urban counties.

Karliner et al (2019) combined data from the San Francisco Mammography Registry and Facility Survey and California Cancer Registry to explore follow‐up times, population vulnerability (defined by race/ethnicity, educational attainment, and English proficiency), system‐based processes, and association with cancer stage at diagnosis in mammography facilities. They found that where population vulnerability was highest, facilities had longer follow‐up times and these facilities also had fewer radiologists, longer biopsy appointment wait times, and less direct patient communication. However, even within these facilities, whites had better outcomes than their nonwhite counterparts. Longer follow‐up time at a facility was also associated with a higher adjusted odds ratio of advanced‐stage cancer at diagnosis.

This body of work in chronic disease raises many questions for health equity researchers and policy makers about health equity methodology and interventions. First, if we begin to use Area Deprivation Index as a factor to assess risk, what do we do to capture the variation within ADI segments—and how much variation is there? In their study, Durfey and colleagues found that the relationship remained significant for both whites and blacks; however, more work is necessary to determine whether this finding is generalizable to health outcomes beyond chronic disease management. Second, with strong associations between food environment and health, what can be done in the health care arena to help patients who are disadvantaged by things outside of their control such as the foods available to them? Is there room for partnerships between federal, state, and local institutions to improve the situation? The authors also highlight the need to consider place when evaluating programs, including the CMS Medicare Diabetes Prevention Program expanded model. Third, Karliner et al's work demonstrates the variation in quality of care across facilities. Time is critical when facing a possible diagnosis of cancer, so what does this research tell us about effective interventions? Do we focus at the systems level, or combine that with an individual‐level approach to empower patients to advocate for themselves within these systems? Is there room for quality measurement of follow‐up time to biopsy? When the consequence of racial and ethnic disparities in site of care is inequality of care, how can public health interventions improve equity?

The next set of manuscripts examine quality of care as it relates to health equity and provide a springboard from which to begin new discussions on population health. In the first one, the authors examine and underexplored aspect of quality of care—one that occurs before patients even step into clinic doors. Leech, Irby‐Shasanmi, and Mitchell (2019) conducted a pilot field experiment to explore the influence of linguistic and name cues on pediatric provider offices’ reports of availability for well‐child visits. Their findings included that auditors giving linguistic and name cues of black patients were less likely to be told that an office was accepting new patients and were more likely to experience both withholding behaviors and misattributions about public insurance, when compared to the control group.

In their work, Lloren and colleagues (2019) offered innovation in quantifying hospital‐specific health outcome disparities that can be publicly reported for use by patients and hospitals. Their work builds on models already used by CMS under pay‐for‐performance programs. Using dual eligibility and racial identity of African American as indicators of social risk, the authors developed and tested a metric intended to be used to target quality improvement efforts and address health equity across populations. Medicare administrative claims data and the Master Beneficiary Summary File (MBSF) were combined, enabling the authors to examine patterns in readmission for acute myocardial infarction, heart failure, and pneumonia among seniors ages 65 and older. The authors demonstrated that a hospital‐specific disparity metric is both feasible and effective at highlighting relative differences in outcomes within hospitals.

In the only manuscript addressing health outcomes of youth, Cook et al (2019) took a close look at prescription patterns before and after the FDA issued the 2004 FDA Black Box Warning on antidepressant use among youth, indicating an increased risk of suicidal thinking, feeling, and behavior among young patients. Although issues around black box warnings have been discussed in the literature at length, the authors approached this from a health equity perspective to identify gaps in practices among racial and ethnic minorities when compared to whites. They found that although the warning did impact prescribing patterns and resulted in lower prescribing habits postwarning, both provider‐ and patient‐level influences played a significant part in the variance in both overall prescribing patterns and disparities in prescribing patterns after the warning, with providers continuing to prescribe the drugs to minority patients at higher rates than whites. This reduction in disparity masked the potential dangers of black and Latino youth continuing their adherence to prescribed antidepressant use in the face of a black box warning.

What are the best next steps to address barriers to a great quality of care and a more equitable system to access care? Leech et al's work suggests that more work is necessary with front line staff to ensure equitable access to care, which is particularly important in the early childhood years. Lloren et al offer a potential metric to assess quality of care disparities in hospitals, both examining within‐hospital and between‐hospital metrics. And finally, Cook et al suggest that there may be an opportunity for policies in the area of individual outreach and patient/caregiver education rather than relying strictly on provider communication. Together, these manuscripts suggest potential points of intervention for future health equity work.

To put patients first, we need to understand the patient experience and satisfaction with care. The next three articles explore equity issues in this area with a keen eye to innovation in data analysis and interpretation. Elliott et al (2019) approached health equity by exploring within‐group variation by language preference as it relates to inpatient care quality and satisfaction. Using data from the 2014‐2015 Hospital Consumer Assessment of Healthcare Providers and Systems (HCAHPS) survey, they was found that non–English‐preferring patients reported poorer scores for inpatient care experience than their English‐preferring counterparts on most of the measures considered, including Communication with Doctors, Communication with Nurses, Responsiveness of Hospital Staff, Communication about Medication, Discharge Information, and Care Coordination. The study found that the most significant difference was for scores in care coordination and non–English‐preferring patients most often attended hospitals with lower overall average patient experience scores.

Research has shown that place matters when it comes to health, and Elliott et al (2019) responded to the place and health literature by considering patient experience across geographic regions. Using data from the 2015‐2016 Medicare Consumer Assessment of Healthcare Providers and Systems (MCAHPS) Survey and 2010 Census data, the authors attempted to disentangle poverty vs racial segregation and each of their relationship with health disparities. They found that counties with higher‐than‐average patient experience scores were also home to reduced black‐white disparities in patient experience scores. But the racial makeup of the county mattered as well, with higher levels of segregation associated with overall level of health care access. Poverty segregation was only significantly associated with getting care quickly, which is unexpected given the literature on poverty and health.

Using data from the 2014‐2015 Nationwide Adult Medicaid Consumer Assessment of Healthcare Providers and Systems (CAHPS) Survey, Martino et al (2019) explored differences in patient experience across racial and ethnic groups and geographic areas. Four composite measures were the focus of this manuscript: getting needed care, getting care quickly, doctor communication, and customer service. The authors found that when compared to whites, American Indians and Alaska Natives (AIAN) and Asians or Pacific Islanders (API) beneficiaries reported worse experiences but blacks reported better experiences. Additionally, they found that beneficiaries in large, urban areas reported worse experiences than others, particularly with regard to access to care.

A considerable segment of the health equity literature focuses on between‐group variation—but here we learn that when we also consider within‐group variation, our understanding of distinctions of health equity issues and approach solutions is enhanced. Elliott's studies reinforce the need for training in cultural competency, linguistically appropriate services, linguistic support, and health literacy, as well as an eye toward geographic variation in patient experience. But health equity issues extend far beyond the walls of health care facilities—we also need to look outside of the health care system in order to better understand our patient populations and barriers to care that may be relevant to health equity. Access to quality care is often thought of as a rural vs. urban issue, but Martino et al's work demonstrates that a focus on access in urban areas may be a fruitful investment as well. The authors pose three theories that contribute to this result: First, that transportation in urban areas may be insufficient to eliminate the barriers to care; second, only lower‐performing health care providers are accessible; or third, utilization management techniques disproportionately disadvantage these Medicaid beneficiaries. The improved scores among blacks in this study may point to the issues raised in Leech, Irby‐Shasanmi, and Mitchell's study exploring the role of language preference.

Finally, research has shown that in the United States, population health can be driven in large part by access to care. Whether health care coverage comes from private or public providers, it is important to consider the impact of such coverage on health and well‐being. The final two manuscripts explore these issues. First, using data from the 2008‐2014 Medical Expenditure Panel Survey, Winkelman, Segel, and Davis (2019) examined costs, utilization, access, and health across racial and ethnic groups to determine what differences Medicaid enrollment made. They found that compared to those respondents who remained uninsured, those who gained Medicaid reported increases in total health care costs and a significant decrease in out‐of‐pocket costs. They also saw evidence of an increase in prescription drug use and reports of a usual source of care, a decrease in foregone care, and significant improvements in severe psychological distress. Lastly, when they examined the data with a health equity lens, comparing outcomes by race and ethnicity revealed significant differences by race and ethnicity in prescription drug costs and total prescription drug fills.

Marton and colleagues (2019), using data from the American Community Survey (ACS), examined coverage disparities across income, race and ethnicity, marital status, age, gender, and with enough precision to consider local and state geographic variation. Their results showed that the predicted impact of full ACA implementation, including Medicaid expansion, on the probability of having any coverage is as high as 22.6 percentage points. The predicted impact of having any coverage when the ACA is implemented without the Medicaid expansion is as high as 9.5 percentage points. Overall, the impact was greater for those with the lowest income, minority populations, younger populations, women, and rural communities.

We at CMS OMH know that we cannot achieve health equity alone—but together, we can learn from all of these studies to gain a better understanding of health disparities so that all of us can move forward in our work to eliminate disparities.

